# Low rate hippocampal delay period activity encodes behavioral experience

**DOI:** 10.1002/hipo.23619

**Published:** 2024-06-05

**Authors:** Markos Athanasiadis, Stefano Masserini, Li Yuan, Dustin Fetterhoff, Jill K. Leutgeb, Stefan Leutgeb, Christian Leibold

**Affiliations:** 1 Fakultät für Biologie, Bernstein Center Freiburg, Albert-Ludwigs-Universität Freiburg, Freiburg, Germany; 2 Computational Neurophysics Lab, Institute for Theoretical Physics, Universität Bremen, Bremen, Germany; 3 Department Biologie II, Ludwig-Maximilians Universität München, Martinsried, Germany; 4 Neurobiology Department, School of Biological Sciences, University of California San Diego, La Jolla, California, USA; 5 Laboratory for Clinical Neuroscience, Centre for Biomedical Technology, Universidad Politecnica de Madrid, Madrid, Spain; 6 Kavli Institute for Brain and Mind, University of California, La Jolla, California, USA; 7 BrainLinks-BrainTools, Albert-Ludwigs-Universität Freiburg, Freiburg, Germany

**Keywords:** adversarial attacks, CA1, CA3, decoding, hippocampus, working memory

## Abstract

Remembering what just happened is a crucial prerequisite to form long-term memories but also for establishing and maintaining working memory. So far there is no general agreement about cortical mechanisms that support short-term memory. Using a classifier-based decoding approach, we report that hippocampal activity during few sparsely distributed brief time intervals contains information about the previous sensory motor experience of rodents. These intervals are characterized by only a small increase of firing rate of only a few neurons. These low-rate predictive patterns are present in both working memory and non-working memory tasks, in two rodent species, rats and Mongolian gerbils, are strongly reduced for rats with medial entorhinal cortex lesions, and depend on the familiarity of the sensory-motor context.

## 1 | INTRODUCTION

The neural representation of working memory content has strongly been influenced by the idea of continuous attractors (Wang, 2001), where a continuous variable that is kept in memory correlates with graded persistent firing rate of neurons. Premotor neural populations have been shown to exhibit such graded persistent activity while encoding continuous eye position (Major & Tank, 2004). Cortical neurons represent the continuous stimulus features in decision tasks by continuous firing rates (Brody et al., 2002; Machens et al., 2005). In the hippocampus, working memory-related activity has been linked to time cells (MacDonald et al., 2011; Pastalkova et al., 2008), in which time would represent a continuous variable that is encoded by population patterns (bumps) that act as continuous attractors.

Time cell activity, however, turned out to not necessarily encode content other than time (Sabariego et al., 2019). Moreover, persistent activity in the hippocampal formation, so far, has mainly been described on a single cell level in-vitro (Egorov et al., 2002).

As an alternative mechanism for short-term memory storage, theoretical models have proposed short-term synaptic plasticity (Leibold et al., 2008; Mongillo et al., 2008), which would not require ongoing high rate activity, however, experimental evidence for these models is still missing. One prediction of synaptic short-term memory, is that access to this memory would correlate with brief periods of selective neuronal activity (Leibold et al., 2008; Mongillo et al., 2008). We therefore were looking into delay period activity of two previously published hippocampal data sets to identify signatures of selective low-rate activity patterns.

First, we were revisiting animals with bilateral lesions of the medial entorhinal cortex (mEC), which show a behavioral deficit in spatial working memory but no overt deficit in time cell activity in the delay period (Sabariego et al., 2019). Since short-term memory is a prerequisite for working memory, we reckoned that mEC lesions might already affect the former and searched for potential impairments of the delay activity in the animals with mEC lesions, which may not be reflected in time cell activity. We, indeed, were able to identify activity correlates of behavioral performance differences between control rats and mEC-lesioned rats (Sabariego et al., 2019): activity from CA3 in control animals was more predictive of the previous behavioral trial than activity from CA3 in mEC-lesioned animals. The informative components of the activity were carried by only few cells that fired few additional spikes.

In addition to the rat data, we also assessed CA1 activity of Mongolian gerbils (Fetterhoff et al., 2021) during a reward consumption period that did not require to maintain working memory and found identical results as for control rats, suggesting that the informative low-rate activity patterns are not working-memory dependent, but may constitute a hippocampal trace of cortical short-term memory processing. Our findings therefore largely rule out the idea that persistent hippocampal activity serves as the basis for short-term memory, but alternatively suggests that informative low rate activity patterns reverberate short-term memory content, either to convey the information to downstream regions, or as spontaneous reflections of the current synaptic or excitability states that may keep the information available in the local synaptic network.

## 2 | MATERIALS AND METHODS

### 2.1 | Electrophysiological data sets

Both data sets included in our analysis have been previously published (Fetterhoff et al., 2021; Sabariego et al., 2019). Detailed descriptions of the experimental methods can be found in the original papers.

**FIGURE 1:**
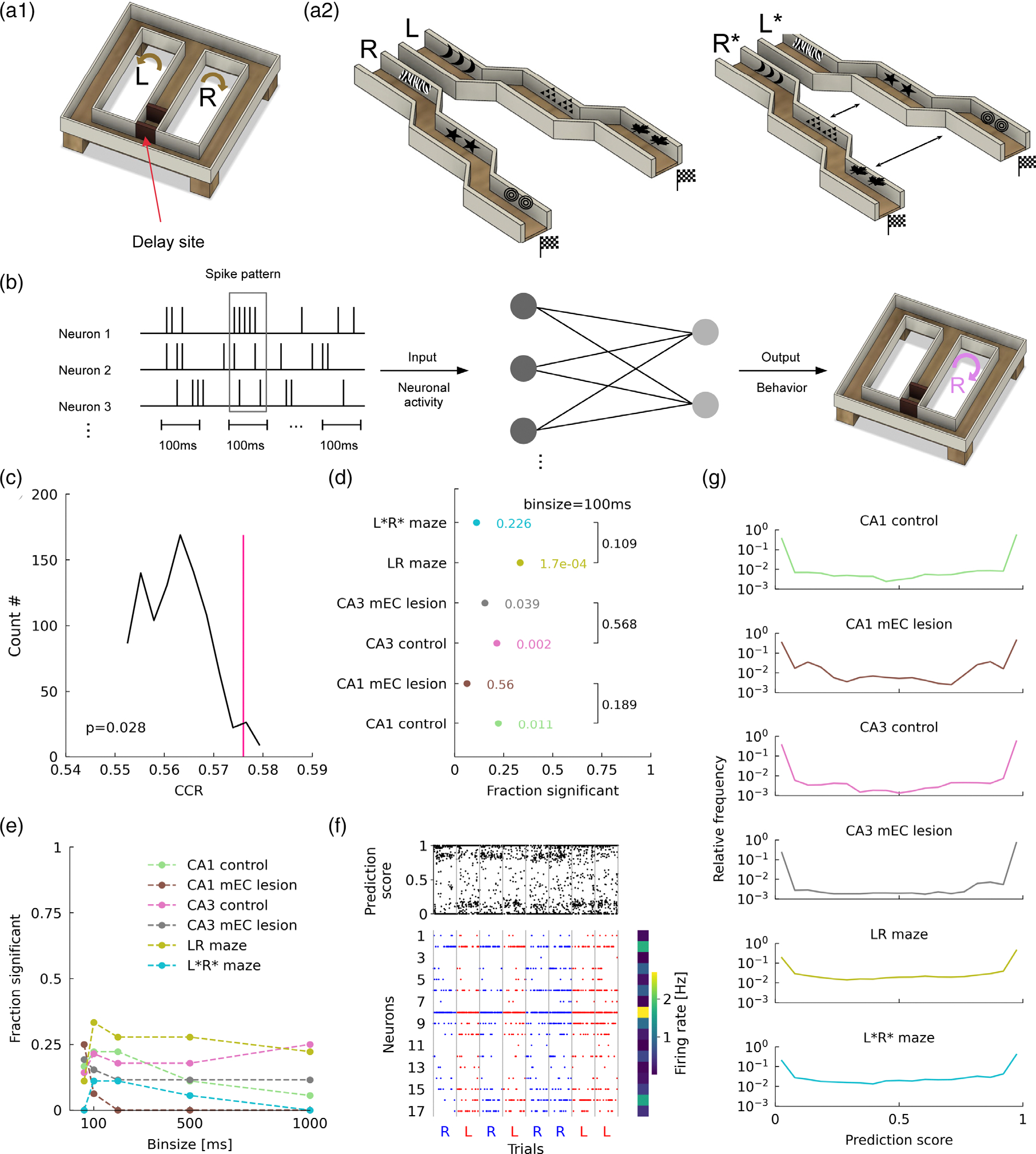
Behavioral paradigms and decoding performance of linear ANN. (a_1_) Rats are trained to alternate between arms of a 8-shaped maze at the end of the middle arm. After each trial rats stayed in a delay site at the beginning of the center stem for 60 s. (a_2_) Mongolian gerbils were trained to run on two mazes, distinguished by left and rightward turns and distinct visual cues placed at the walls of each corridor (middle). Subsequently the gerbils ran through previously unseen environments where the visual cues are flipped between the two mazes (right). (b) Schematic of the decoding process. Time-binned population vectors were used as input to a linear neural network that is trained to predict binary class labels (R: right; L: left) as an output. (c) Correct classification rate (CCR; pink) for an example CA1 recording from a control rat (rat 3903/day 1/session 2/5 left trials/4 right trials) and distribution of CCRs for label shuffles (black). Despite being small, the CCR is significant (*p* value as indicated; 1000 shuffles; 100 cross validation iterations; number of test patterns (100 ms) bins used in cross validation: 2700). The number of trials and test patterns for all conditions is summarized in Table 1ii,iii. (d) Fraction of sessions for which the permutation test from (c) was significant for each of the six groups of experiments (p values from binomial tests; CA1 control: 4/18; CA3 control: 6/28; RL maze: 6/18). mEC lesions in rats and unfamiliar arrangement of visual landmarks in gerbil data lead to decreased decoding (*p* values as indicated; CA1 mEC lesion: 1/16; CA3 mEC lesion: 4/26; L*R* maze: 2/18 Chi squared test for homogeneity, rat CA1: $\chi^{2}=1.723,\;n_{1}=4,\;n_{2}=1$; rat CA3: $\chi^{2}=0.326,\;n_{1}=6,\;n_{2}=4$; gerbil CA1: $\chi^{2}=2.571,\;n_{1}=6,\;n_{2}=2$). (e) The fraction of significantly decodable sessions decreases for larger time intervals L in CA1 data sets but remains at a constant level in CA3 data sets. (f) Prediction score (PS) for an example session from CA1 of a rat with mEC lesion (Rat 3928/day 2/session 1) using L=100ms time intervals (top) and spike raster plot (bottom) from a 60 s delay period succeeding left- and right-ward trials (red and blue, respectively). Color bar indicates that mean firing rates vary substantially across neurons. (g) Distributions of the PS for all groups of experiments only exhibit a small bias towards 1.

In brief, 15 male Long Evans rats were trained on the spatial alternation task (Sabariego et al., 2019) and randomly assigned to one of two groups, a group with nearly complete NMDA lesions of the medial entorhinal cortex (n=7) and a control group (n=8). After about 9 weeks of recovery, both groups of animals were implanted with tetrodes that were lowered until the CA1 or CA3 region. The behavioral task was performed using an 8-shaped maze (Figure 1a1). Rats were trained until the performance reached 90% correct trials on 2 of 3 consecutive days. After that 30 trials with 60 s delay were performed daily for each rat, for 14 days.

From the virtual reality task (Fetterhoff et al., 2021), we obtained data from six male Mongolian gerbils (Meriones unguiculatus) with tetrodes implanted to dorsal hippocampal CA1. Gerbils were trained to the task of running on a 620 cm long linear track consisting of three linear hallways separated by two 45 corners. The animals were initially introduced on the two original maze types: R and L, each containing two right or two left turns and, each containing different images (Figure 1a2). After learning original image-turning direction combinations, images were swapped in the middle and the last hallways (L* and R* mazes). During recording sessions, 20 randomly-ordered original mazes were presented before 20 randomly ordered swapped mazes. The VR system is described in greater detail in Thurley et al. (2014).

### 2.2 | Population vectors

Spikes of all N neurons recorded during the delay phases of a session are time-binned in t=1,…,T intervals with bin size L. The number T of time bins is also called the size of the data set. Neurons without any spike are excluded from the data set (see Table 1i) resulting in population vectors ${\vec{x}}_{t}={\left(x_{t}^{\left(1\right)},\ldots ,x_{t}^{\left(N\right)}\right)}^{⊤}$. Each of the neurons is converted to the standardized space, by subtracting the mean neuron activity in a session and then dividing the difference by the standard deviation of the neuron activity,

(1) $${\vec{x}}_{t,n}=\frac{{\vec{x}}_{t,n}\;-\;\bar{{\vec{x}}_{*,n}}}{\sigma_{{\vec{x}}_{\text{*},n}}}$$

Each of the patterns ${\vec{x}}_{t}$ is assigned a label $l_{t}=\pm 1$ according to the binary behavioral experience in the trial this pattern is obtained from. In our data sets, these binary labels distinguish rightward from leftward turns.

### 2.3 | Artificial data

#### 2.3.1 | Linear separation task

We generate a linearly separable data set of t=1,…,T vectors

(2) $${\vec{x}}_{t}^{\left(\pm \right)}=(\pm )\frac{d}{2}\vec{w}+{\vec{\xi }}_{t}^{\left(\pm \right)}$$

with labels $l_{t}=\pm 1$. Here, d≥0 denotes the signal along the ground truth direction ${\vec{x}}_{t}^{\left(\pm \right)}$ that is added (subtracted) to normal i.i.d. random vectors ${\vec{\xi }}_{t}^{\left(\pm \right)}$. The dimension n of the vectors ranges between 2 and 100. The sparseness s=1/n of the weight vector indicates only one active dimension, and the signal strength d varies between 0 and 10.

#### 2.3.2 | Artificial spiking model

To mimic random spiking activity we simulate n homogeneous Poisson processes with density λ (varying between 0.05 and 0.2) and construct t=1,…,T population vectors from time bins of size 1 with balanced random labels $l_{t}=\pm 1$.

A ground truth weight vector $\vec{w}$ is then applied to a small subset $T^{\prime }=\left(1-p_{\text{fail}}\right)T$ of the available vectors from the positive subset (l=+1):

(3) $${\vec{x}}_{m}^{\left(+\right)}={\vec{x}}_{m}^{\left(+\right)}+\vec{w}.$$

The sparseness s of the binary weight vector varies between 10% and 30%. The dimensions are varied between 10 and 50.

### 2.4 | Decoding

We train a binary classifier to distinguish the binary behavioral choices. Our specific choice of the classifier is a *linear* neural network, implemented in *PyTorch* (Paszke et al., 2019). The network consists of an input layer $\vec{x}$, with one node for each of the active neurons we attempt to decode, and an output layer $\vec{O}$ with two nodes, each dedicated to one of the binary labels. The output is computed using the softmax function

(4) $$\vec{O}=\vec{\sigma }(W\vec{x}),\;with\;\sigma (\vec{h}{)}^{\left(k\right)}=\frac{e^{h_{k}}}{e^{h_{1}}\;+\;e^{h_{2}}},k=1,\;2$$

The training of the classifier minimizes the cross entropy loss function in the space of the 2×N weight matrices W. Typically, supervised training occurs for 1000 consecutive epochs, with a learning rate 0:001 (see Paszke et al., 2019).

In order to ensure a less biased estimate of the model performance and to avoid overfitting, we employ a 2-fold crossvalidation process during which we generate 100 random separations into training and a testing subset of equal size T/2, in which the ratio between the two labels is kept as in the full data set. Applying the classifier on the test data in each of the 100 random separations yields a fraction of correct classifications. As correct classification rate (CCR) we define the mean of these 100 fractions.

We repeat the decoding for 1000 random shuffles of labels and thereby obtain 1000 CCRs (each averaged on 100 random separations into test and training sets) from which we construct the null distribution that is used to assign a *p* value to the decoding performance as the percentile of the real CCR.

### 2.5 | Adversarial attacks

To identify the separating hypersurface we ran two repetitions of the fast gradient sign method (FGSM) attack on each data point. The FGSM attack takes advantage of the gradient descent optimization of a neural network, and is executed via the Python-based package *Foolbox* (Rauber et al., 2020), which provides reference implementations of a variety of published state-of-the-art adversarial attacks (Goodfellow et al., 2014).

The attack maps each population vector ${\vec{x}}_{t}$ onto a different vector $A\left({\vec{x}}_{t}\right)$ which is moved to the hemispace opposite to the separating hypersurface. We apply the attack process twice since then the resulting vectors ${\vec{a}}_{t}=A\left(A\left({\vec{x}}_{t}\right)\right)$ faithfully sample the classification boundary. After the two attacks a population vector ${\vec{x}}_{t}$ is thus associated with a vector ${\vec{a}}_{t}$ that is supposed to be proxy for the closest position on the separating hypersurface.

In order to avoid overfitting of the decision boundary, we repeat the computation of attack vectors ${\vec{a}}_{t}$ 100 times using random subsamplings of the data set of size T/2 keeping the ratio of labels as in the full data set.

### 2.6 | Most informative directions

As most informative direction (MID) we define the direction in the space of population vectors ${\vec{x}}_{t}$ that is orthogonal to the decision boundary of the classifier. Since in general the decision boundary can be non-linear, MIDs are local and thus we expect that multiple MIDs can occur for any given dataset. It also needs to be noted that MIDs are a property of the data set and not the classifier. Thus even if we use a linear classifier to approximate parts of the (potentially non-linear) decision boundary, we may obtain multiple MIDs, depending on which part of the boundary is best matched by the current subsampling of the data set.

To obtain the orthogonal direction at one attack vector location ${\vec{a}}_{t}$, we compute a set of difference vectors ${\vec{d}}_{t,k}={\vec{a}}_{t}-{\vec{v}}_{t,k}$ (Figure 3a, green arrows) with ${\vec{v}}_{t,k}=\left\{{\vec{a}}_{t^{\prime }}|r_{t}<D\left({\vec{a}}_{t},{\vec{a}}_{t^{\prime }}\right)<R_{t}\right\}$ denoting subset of attack vectors in a ring-shaped vicinity of ${\vec{a}}_{t}$. As a distance function D we use the Euclidean distance, with $r_{t}=0.02\;{max}_{t^{\prime }}\;D\left({\vec{a}}_{t},{\vec{a}}_{t^{\prime }}\right)\;$ In all and $R_{t}=0.35{\;max}_{t^{\prime }}\;D\left({\vec{a}}_{t},{\vec{a}}_{t^{\prime }}\right)$.

The MIDs are then obtained by searching the directions ${\vec{n}}_{t}$ that minimize the squared scalar product to the distance vectors, i.e.,

(5) $${\vec{n}}_{t}={argmin}_{{\vec{n}}_{t}}{\left({\vec{n}}_{t}\cdot \sum_{k}\;\;{\vec{d}}_{t,k}\right)}^{2}\;\text{with}\;|{\vec{n}}_{t}|=1.$$

The minimization is equivalent to finding the Eigenvector ${\vec{n}}_{t}$ of the matrix $\sum_{k}\;{\vec{d}}_{t,k}\cdot {\vec{d}}_{t,k}^{⊤}$ with the smallest Eigenvalue. Since the minimization problem in Equation (P 5) is symmetric regarding multiplication with −1, we always choose ${\vec{n}}_{t}$ pointing into the +1 hemispace.

Finally, we apply the density-based spatial clustering algorithm (DBSCAN from the Python package *scikit learn* (Pedregosa et al., 2011)), to all vectors ${\vec{n}}_{t}$ and to obtain C cluster representatives ${\vec{n}}^{\left(c\right)},c=1,\ldots ,C$, which we call MIDs. To generate robust estimates of the MIDs, we repeat the procedure 100 times on subsampled data sets of size T/2 (keeping the ratio of labels) and derive the two quality measures *amount* $\alpha^{\left(c\right)}$ and *consistency* $\chi^{\left(c\right)}$: As amount we use the fraction of attack vectors ${\vec{a}}_{t}$ whose normals ${\vec{n}}_{t}$ are assigned to the cluster c (averaging over all subsamplings). As consistency $\chi^{\left(c\right)}$ we denote the fraction of all subsamplings which end up in finding the same cluster c. To identify whether MIDs from two subsamplings are in the same cluster, we apply the clustering algorithm DBSCAN to all identified MIDs. The performance of DBSCAN can be adjusted by two main parameters. The first parameter is the maximum distance between two vectors in the same cluster, and is set to 0:25 unless mentioned otherwise. The second parameter is the minimum number of samples within a cluster to not be considered as noise, and is set to 3% of the dataset (Ester et al., 1996).

### 2.7 | Bias correction

MIDs are vectors orthogonal to the decision boundary and thus in order to compute the overlap $q_{t}^{\left(c\right)}$ between MID c and a specific pattern ${\vec{x}}_{t}$, we first subtract the bias ${\vec{y}}_{t}={\vec{x}}_{t}-{\vec{b}}_{t}$ and then compute the scalar products $q_{t}^{\left(c\right)}={\vec{n}}^{\left(c\right)}\cdot {\vec{y}}_{t}$. The bias vectors ${\vec{b}}_{t}$ are obtained in every time bin as the center of gravity of the ${\vec{a}}_{t}$ vectors the MID is composed of.

### 2.8 | Relevant time bins

To identify whether an activity pattern ${\vec{x}}_{t}$ reflects a certain MID, we generate a Null distribution for the overlaps $q_{t}^{\left(c\right)}$ by 1000 random shuffles of the neuron indices. Relevant time bins for MID c are those in which $q_{t}^{\left(c\right)}$ exceeds the upper 97.5%-tile or falls below the lower 2.5%-tile.

### 2.9 | Local field potential analysis

In all recordings from control rats, we selected for the LFP analysis the channels with highest theta power among the tetrodes which were located in the same brain region (CA1 or CA3). Different oscillation bands were extracted by applying a FIR bandpass filter (theta: 6–11 Hz, slow gamma: 30–50 Hz, mid gamma: 55–90 Hz, fast gamma: 95–140 Hz, ripples: 150–250 Hz) based on a Hamming window. Bandpass filtered signals were Hilbert-transformed and the mean square Hilbert-amplitude in a time bin of length L was used as an estimate for short-term power analysis.

## 3 | RESULTS

To explore the information content of hippocampal activity during waiting periods, we examined two data sets in which animals performed different behavioral tasks. In a first data set, two groups of rats (with and without bilateral mEC lesions) were trained on a spatial alternation task with a variable waiting period between trials, in which they needed to maintain a working memory of their previous behavioral choice (Figure 1a1). Here, we only focused on sessions with 60 s long delay periods. Previously, it was shown that in animals with mEC lesions task performance is degraded (Sabariego et al., 2019) but it remained unclear whether this behavioral finding is reflected in hippocampal activity during the delay period. In a second data set Mongolian gerbils were trained to run on two mazes in virtual reality (distinguished by left and rightward turns and a turn-direction specific set of visual cues; Figure 1a2), that were selected in random order (such that no information about the future can be represented in inter trial intervals and animals had no requirement of working memory), and had a 20 s pause between trials during which animals received a reward (Fetterhoff et al., 2021). We compared two types of virtual mazes, a familiar configuration of visual cues in which the animals have been trained on the task, and a “swapped” maze in which visual cues are presented in association with the other turn direction introducing sensory conflicts, while the animals kept performing the same task.

### 3.1 | Decoding performance

In the original analysis for the delay activity in the rat data sets (Sabariego et al., 2019), a linear (support vector) classifier was unable to distinguish whether preceding trials had left and right turns when population vectors were constructed with L=1s binning. Here, we repeated the analysis with a shorter time interval L=100ms matching the typical duration of population bursts and a linear neural network to predict the left/right label of the trial preceding the delay period in all six groups of experiments (Figure 1b). Correct classification rates (CCR) were slightly but significantly above chance (see example in Figure 1c) in a fraction of rat recording sessions that exceeded randomness (according to binomial tests, see figure caption) except for CA1 recordings from rats with mEC lesions (Figure 1d). Original virtual reality mazes could also significantly be decoded from delay activity (Figure 1d, LR maze), but swapped mazes with sensory conflicts could not (Figure 1d, L*R* maze). Since we observe predictions of previous trial labels, even in the gerbil data set without a working memory task, we reason that the activity does not specifically reflect working memory. Nevertheless the activity may underlie the establishment of working-memory although differences between mEC-lesioned and control rats do not yet reach significance at this level of analysis.

The fraction of significant sessions was generally highest for L=100ms intervals (except in CA1 recordings from MEC-lesioned animals, where the fraction of significant sessions was maximal for 50 ms binning) and decreased with larger bin sizes (Figure 1e; except for CA3 in control rats) indicating that, at least in CA1, the information about the previous turning direction is mostly carried by short-term correlations. The finding that CA3 activity even for L=1s is significantly predictive contradicts previous reports in (Sabariego et al., 2019) and arise due to different preprocessing (scaling). We ruled out that differences are induced by the use of a neural network as compared to a linear support vector machine showing similar performance in Figure S1. Further insight into the predictive activity patterns (see Section 3.3), will further explain differences between CA1 and CA3 results. The fact that we can successfully decode only a small but significant fraction of sessions already hints at only a few neurons being informative. Thus, the chance that any given random subsample of neurons picked up by the tetrode recordings in a session contains informative neurons will be low.

To better understand what activity features the classifiers use to distinguish past experiences, we first computed a prediction score (PS) for every time bin. The PS measures the fraction of repetitions in which a population vector from a particular time bin yielded a correct prediction during testing (see Section 2). One representative example session (Figure 1f) reflects a general directional bias (here “left”) of the classifier, that is, the prediction outcomes tend to favor the label “left” independent of the real trial label if no information seems available in the spiking pattern. The above chance performance of the classifier on average (Figure 1g) is reflected in PS distributions with a peak at 1 only slightly exceeding the peak at 0.

One possible explanation for the low average CCR values and the small bias in PS that is in accordance with the general increase in prediction for lower bin sizes L is to assume that the informative neural signatures occur only in few brief time intervals. A natural guess would therefore be to investigate the association between intervals of high PS and awake sharp-wave ripple (SWR) events, since they are of about 100 ms length, are generally thought to support planning (Jadhav et al., 2012; Shin et al., 2019), and the incidence rates are affected by functional mEC inputs (Chenani et al., 2019).

**FIGURE 2:**
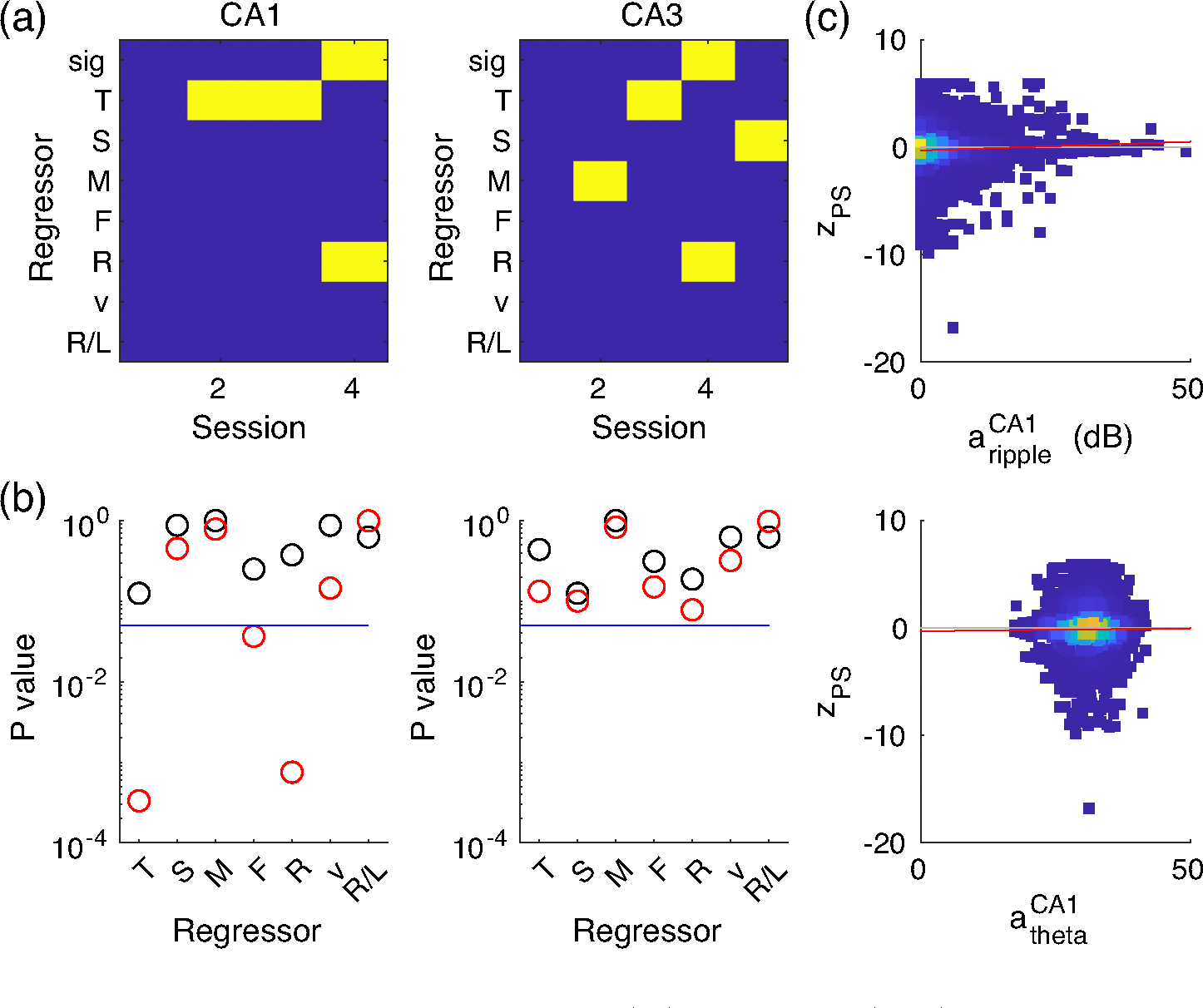
Lack of clear correlation between z-scored prediction score (PS) and LFP power (in dB) for the nine significant control sessions. (a) Significance (*p*-value < .05: yellow; *p*-value > .05: blue) of general linear model fits of the *z*-scored PS using the regressors theta power (T, 6–11 Hz), slow- (S, 30–50 Hz), mid- (M, 55–90 Hz), fast-(F, 95–140 Hz) gamma, and ripple (R, 150–250 Hz) power, speed (v), and label (R/L) for CA1 (left) and CA3 (right) recordings for control rats. Significance for the whole model fit (sig) is obtained from *F*-statistics, significance for the *β* values from *T*-statistics. Numerical values for test statistics and *p* values are provided in Table 2. (b) *p* values for fits to pooled data (*T*-statistics, red) and Wilcoxon tests on regression coefficients (*β*-values) of the individual sessions being different from zero (black). (c) Scatter (density) plots for *z*-scored PS versus regressors theta power (bottom) and ripple power (top) with regression line (red).

We tested this conjecture for the control data sets from rats (CA1 and CA3) performing a spatial working memory task, by correlating the local field potential (LFP) power in different frequency bands with the prediction scores of the classifier in 100 ms bins. We, however, did not find any consistent correlation between spectral bands and prediction (Figure 2a, Table 2) by standard multilinear regression. Only 1 of 4 significant CA1 session and 1 of 5 significant CA3 session showed overall significant linear relation (ANOVA) with 3 of 9 (=4 CA1 + 5 CA3) individual tests showing significance in the theta band and 2 of 9 in the ripple band. Wilcoxon signed-rank tests for non-zero regression weights across sessions (black circles in Figure 2b) did show no significant results, suggesting that overall dependencies of prediction scores on LFP must be weak. This conclusion was further corroborated by inconsistent significance of correlations between LFP power and prediction scores when data were pooled over all sessions. Pooled CA1 prediction scores, were significantly modulated with ripple power, but not the pooled CA3 prediction scores (red circles in Figure 2b). We visualized the best candidate correlations (CA1 theta and ripple) as scatter plots, which revealed that the significant linear regression of the pooled prediction scores may only explain a negligibly small part of the variance (Figure 2c, Table 3). With this observed lack of clear correlation we rule out that successful decoding mostly relies on SWR or any other LFP-related activity pattern.

### 3.2 | Most informative directions

**FIGURE 3:**
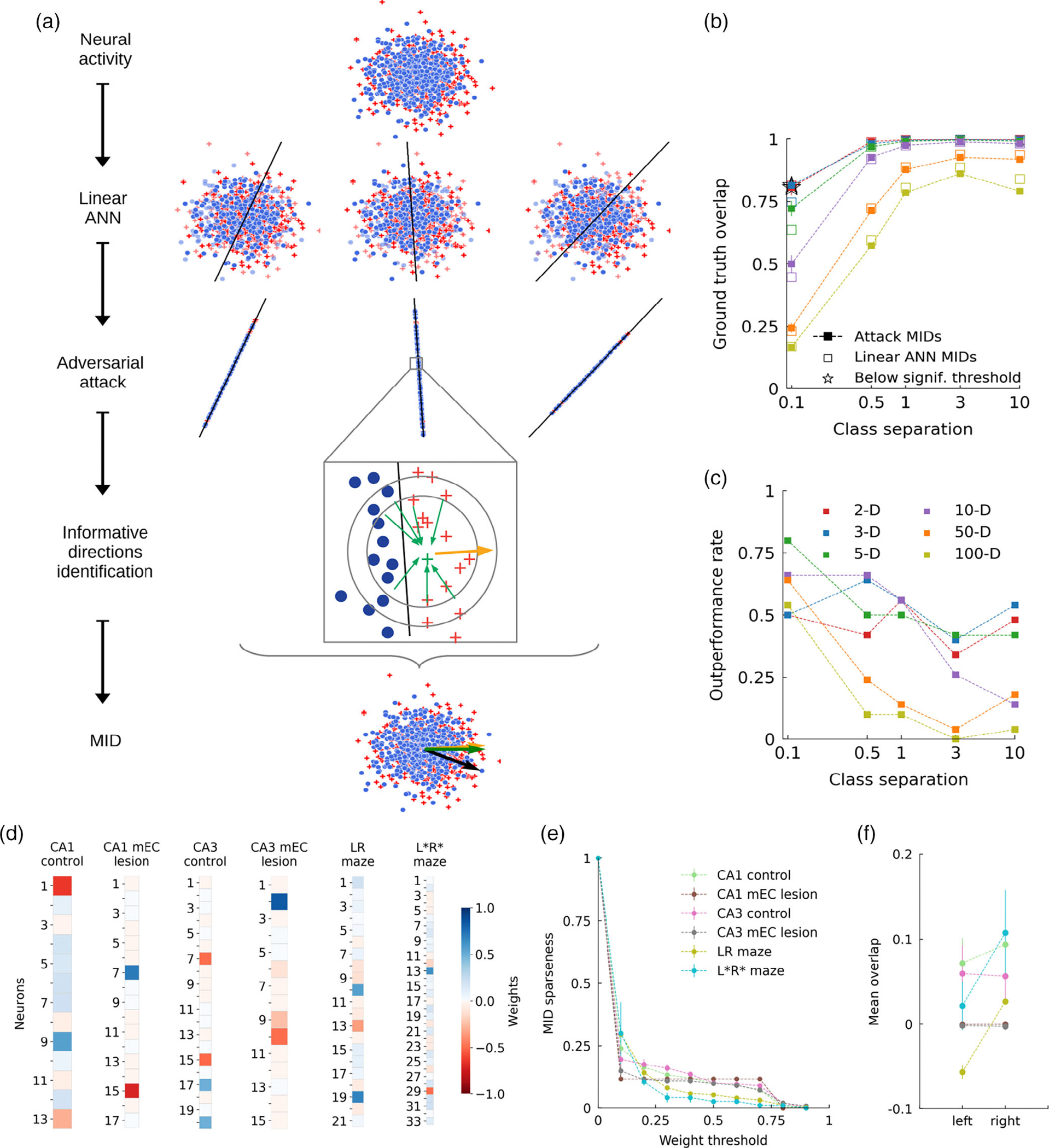
Most informative directions (MIDs). (a) Illustration of the MID identification process (Section 2). A linear classifier is trained using a 2-fold cross validation scheme. Adversarial attack methods are employed to move data points close to the decision boundary. MIDs are then identified by clustering (DBSCAN) locally orthogonal vectors (gold). Results are grouped, sorted and evaluated over all cross validation iterations (100 bootstraps). (b) Overlap of MIDs and ANN weights with the ground truth for a linear classification task on synthetic data (n=50); see Section 2. MIDs outperform the linear ANN weight for high dimensionality and high degrees of by-class overlap. Stars indicate results below significance threshold. The feature space is color coded. (c) Rate at which MIDs outperform the linear ANN weight across repetitions (n=50). (d) MIDs from example sessions for all groups of experiments (CA1 control rat 3906/day 1/session 2;CA1 mEC-lesioned rat 3928/day 2/session 1; CA3 control rat 3958/day 3/session 2; CA3 mEC-lesioned rat 3903/day 1/session 2;RL maze gerbil 2783/day 1; R*L* maze gerbil 2784/day 3). Saturated colors indicate neurons which contribute more strongly to the decision boundary. (e) Fraction of MID weights (sparseness) exceeding a certain threshold. (f) Mean overlaps of population vectors with MIDs for “left” and “right” labeled trials.

To directly identify the neuronal basis of the prediction scores of the classifier and to be able to separate effects of classifier bias from informative neural activity, which both affect prediction scores in Figure 1f we were seeking for an approach to visualize the decision boundary of the classifier, that is, to identify the neural ensembles that are specific to the previous experience of the animal. To do so, we applied adversarial attack techniques from machine learning (see Section 2) (Goodfellow et al., 2014; Rauber et al., 2020) that move the population vector constructed from a specific time point to a position close to the classification boundary (Figure 3a). In brief, attack methods move correctly classified data points towards the nearest decision boundary of a classifier to achieve misclassification, and, in doing so, help understanding problems of generalization and potential errors for out-of-distribution samples. Here, we use iterated attacks to move data points as close as possible to the boundary to sample it. Having obtained such a set of data points at the boundary, we then constructed most informative directions (MIDs, orange vector in Figure 3a) as clusters of orthogonal vectors to the boundary. The method outperforms estimating the weight vector by bootstrapping the training process on multiple subsamplings for low levels of class separation (Figure 3b,c).

Examples for MIDs from all six data sets are shown in Figure 3d, indicating only few active neurons (saturated colors) to contribute to the decision of the classifier. Varying the weight threshold to obtain heuristic sparseness estimate reveals that only about 20% of the neurons (that were active in the delay period) may contribute to the classification performance (Figure 3e).

To test whether the obtained MIDs indeed identify functionally relevant dimensions, we assigned overlap values $q_{t}^{\left(c\right)}$ with all the MIDs (identified by c) to the population vectors (identified by time index t). If the sign of q correlates with the decision performance, we would consider the MID informative. However, we only find such a sign change to occur in the control gerbil data set (Figure 3f), suggesting that averaging over all time bins probably blurs the signal and thus proceeded with restricting our analysis to only those “relevant” time bins which we suspect to be most informative.

### 3.3 | Low rate relevant time bins

**FIGURE 4:**
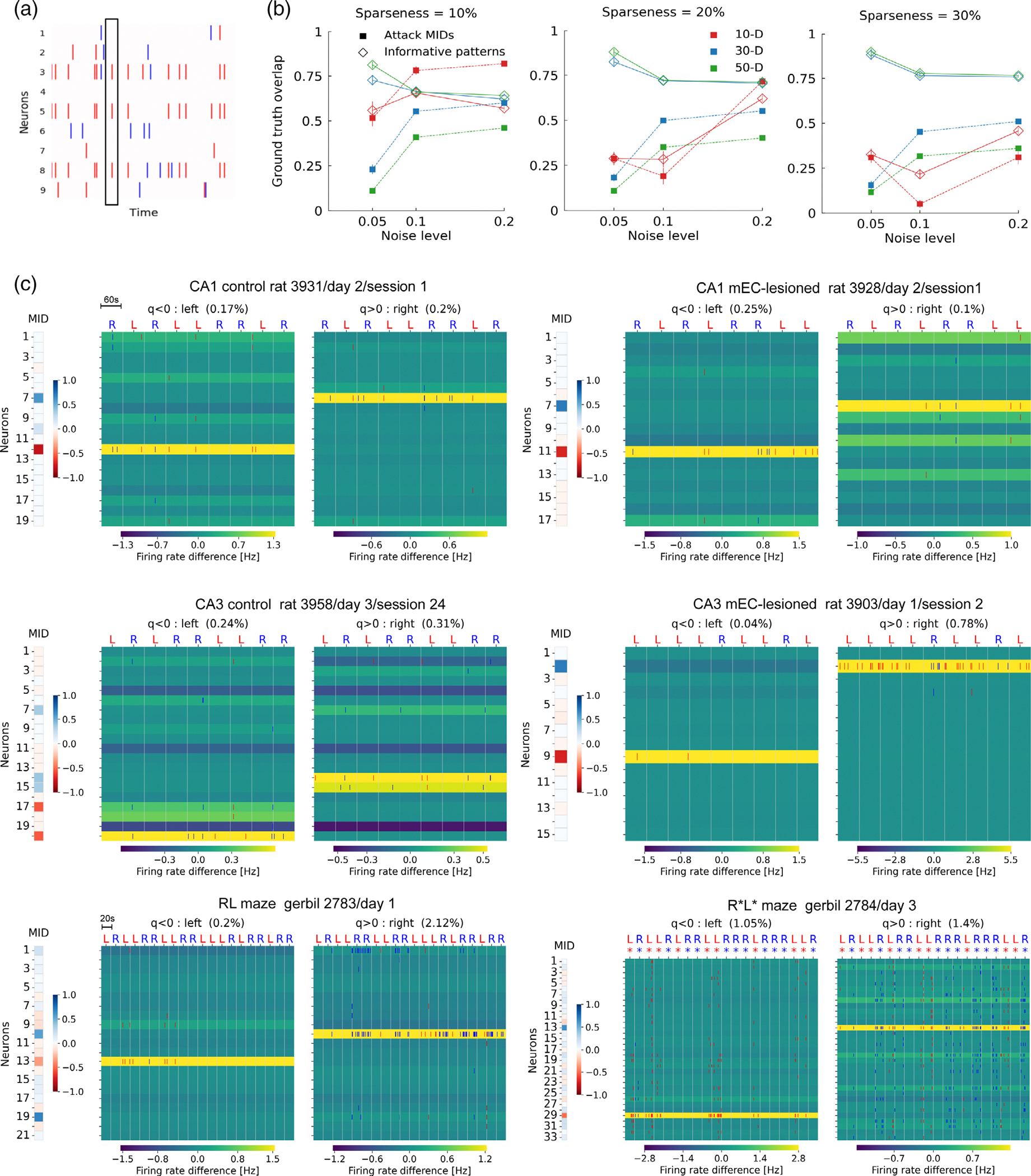
Relevant time bins. (a) Simulated spiking activity for a binary behavioral task (see Section 2). Dotted line indicates a population vector. Blue and red colors indicate behavioral labels (R/L). Relevant time bins are defined by populations vectors with significant overlap to a MID. (b) Overlap of MIDs (square markers) derived from synthetic spiking data (see Methods) and patterns from relevant time bins (diamond markers) with the ground truth, while varying noise, sparseness, and neuron number (colors). Patterns from relevant time bins outperform MIDs except few (~10) cells at high background noise levels (red traces in left panel). (c) MIDs (left) and corresponding spike raster plots (right) of concatenated delay periods for an example session per condition (CA1 control: rat 3931/day 2/session 1; CA1 mEC-lesioned rat 3928/day 2/session1; CA3 control rat 3958/day 3/session 2; CA3 mEC-lesioned rat 3903/day 1/session 2; RL maze gerbil 2783/day 1; R*L* maze gerbil 2784/day 3). Only spikes are shown that occur in relevant time bins. Spikes are colored (red/blue) according to trial labels. The background colors indicate the differences in firing rate between relevant and non-relevant bins of the individual neurons. Percentages on top reflect fractions of bins identified as relevant.

To identify relevant time bins, we compared the overlap values q with the shuffle distribution (see Section 2; Figure 4a,b) to find above-chance overlap with the MID. Time bins for which $q_{t}$ was below the 2.5 percentile of the shuffle distribution (significantly negative overlap) were considered to be predictive for “left” labels, time bins for which $q_{t}$ was above the 97.5 percentile (significantly positive overlap) of the shuffle distribution were considered to be predictive for “right” labels. Figure 4c depicts six examples of spike patterns from the relevant time bins. These examples are typical in that the firing rate in relevant bins of mostly only one neuron considerably exceeds its firing rate in the non-relevant bins, and this neuron gets the largest load of the MID in positive (right) and negative (left) direction. We also observe general modulations of firing rates across trials with some trials having increased activity in all neurons.

**FIGURE 5:**
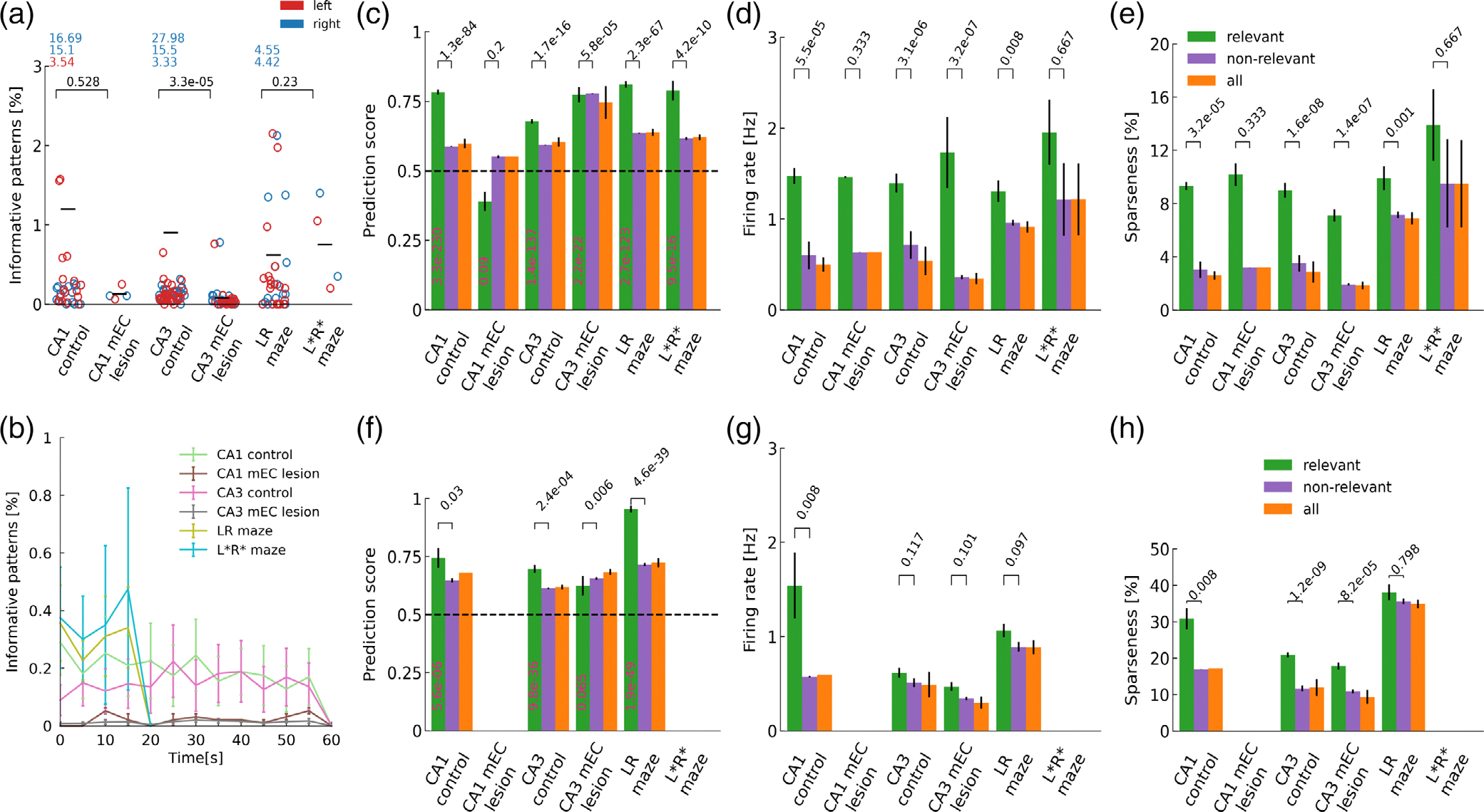
Population statistics from relevant time bin. (a) Mean percentage of relevant time bins for each condition with respect to “left”-(red) and “right”-ward (blue) turns. Outliers are printed as numbers on top. mEC-lesioned rats showed significantly fewer relevant time bins (Mann–Whitney U test; p values indicated; rat CA1: $U=23.5,n_{1}=18,n_{2}=2$; rat CA3: $U=487.5,n_{1}=30,n_{2}=19$; gerbil CA1: $U=8.5,n_{1}=19,\;n_{2}=2$). (b) Distribution of relevant time bins across the delay periods for all sets of experiments does not show a trend towards early or late periods. (c) The prediction scores for relevant time bins are significantly above chance for all sets of experiments except for the CA1 in mEC-lesioned animals (Wilcoxon test; see Table 4i). Prediction scores of relevant time bins are significantly larger than the non-relevant ones in all sets of experiments apart from the mEC-lesioned animals (Mann–Whitney *U* test; see Table 4ii). (d) Significantly higher firing rates are observed in relevant vs. non-relevant time bins for all sets of experiments apart from the CA1 of lesioned rats and the unfamiliar L*R* mazes in gerbil CA1 (Mann–Whitney *U* test, see Table 4iii). (e) Significantly higher fraction of active cells are observed in relevant vs. non-relevant time bins for all sets of experiments apart from the CA1 from lesioned rats and the unfamiliar L*R* mazes in gerbils (Mann–Whitney *U* test, see Table 4iv). (f) For longer time bins (1 s), the prediction scores of relevant time bins are significantly above chance for all sets of experiments except for the CA1 in mEC-lesioned animals and L*R* mazes where no relevant bins can be identified (Wilcoxon test; see Table 4v). Prediction scores of relevant time bins are significantly larger than the non-relevant ones in all sets of experiments apart from the mEC-lesioned animals and L*R* mazes where no relevant bins can be identified (Mann–Whitney *U* test; see Table 4vi). (g) Significantly higher firing rates are observed in relevant versus non-relevant time bins only for the CA1 of control rats when longer time bins (L=1 s) are used (Mann–Whitney *U* test, see Table 4vii). (F) Significantly higher fraction of active cells are observed in relevant versus non-relevant time bins for the CA1 and CA3 control rats as well as the CA3 lesioned rats (Mann–Whitney *U* test, see Table 4viii). Firing rate differences between relevant and non-relevant bins are generally less pronounced for larger bin sizes.

These examples are also typical, in that only a small fraction of time bins turned out to be relevant in general (Figure 5a) with most relevant bins (2.4%) in CA1 data from control rats. Rats with mEC lesions showed particularly low fractions of relevant bins with the difference between control and lesioned animals reaching significance only for CA3 recordings (Mann–Whitney *U* rank test). This finding suggests the mEC supports the expression of brief periods of informative delay period activity that, at least in CA3, may reflect working memory performance. The sparsely interspersed relevant time bins thereby occur at similar rates across the delay period in all analysis groups (Figure 5b).

Despite PS over all time bins only had a tiny bias towards 1, prediction scores in the relevant bins are very clearly and significantly above chance (Wilcoxon test; see Table 4i) for all data sets except CA1 recordings from lesioned rats. Also PS in relevant bins were significantly larger (Mann–Whitney *U* rank test; see Table 4ii) than in non-relevant bins except for the data sets from mEC-lesioned animals (Figure 5c). These findings indicate that MIDs provide a handle for identifying predictive neural activity except in the two data sets from lesioned animals, possibly because there are just too little relevant time bins (Figure 5a).

The number of spikes contributing to the above chance prediction is very small as indicated by firing rate (Figure 5d) and sparseness (Figure 5e), but most data sets exhibit significantly increased firing rate and fractions of active cells in relevant bins as compared to non-relevant bins (Mann–Whitney *U* rank test; see Table 4iii,iv), indicating that indeed few brief intervals of slightly enhanced activity carry the behavioral information. In this context, we also revisited the unexpectedly large predictability of CA3 activity in long time bins of L=1s (Figure 5f–h, Table 4v–viii), and found a lower firing rate difference between relevant and non-relevant time bins. This indicates that predictive activity patterns in CA3 seem to be dispersed over longer time periods (seconds) than in CA1 (milliseconds).

### 3.4 | Relevant patterns activity is unrelated to spatial tuning

**FIGURE 6:**
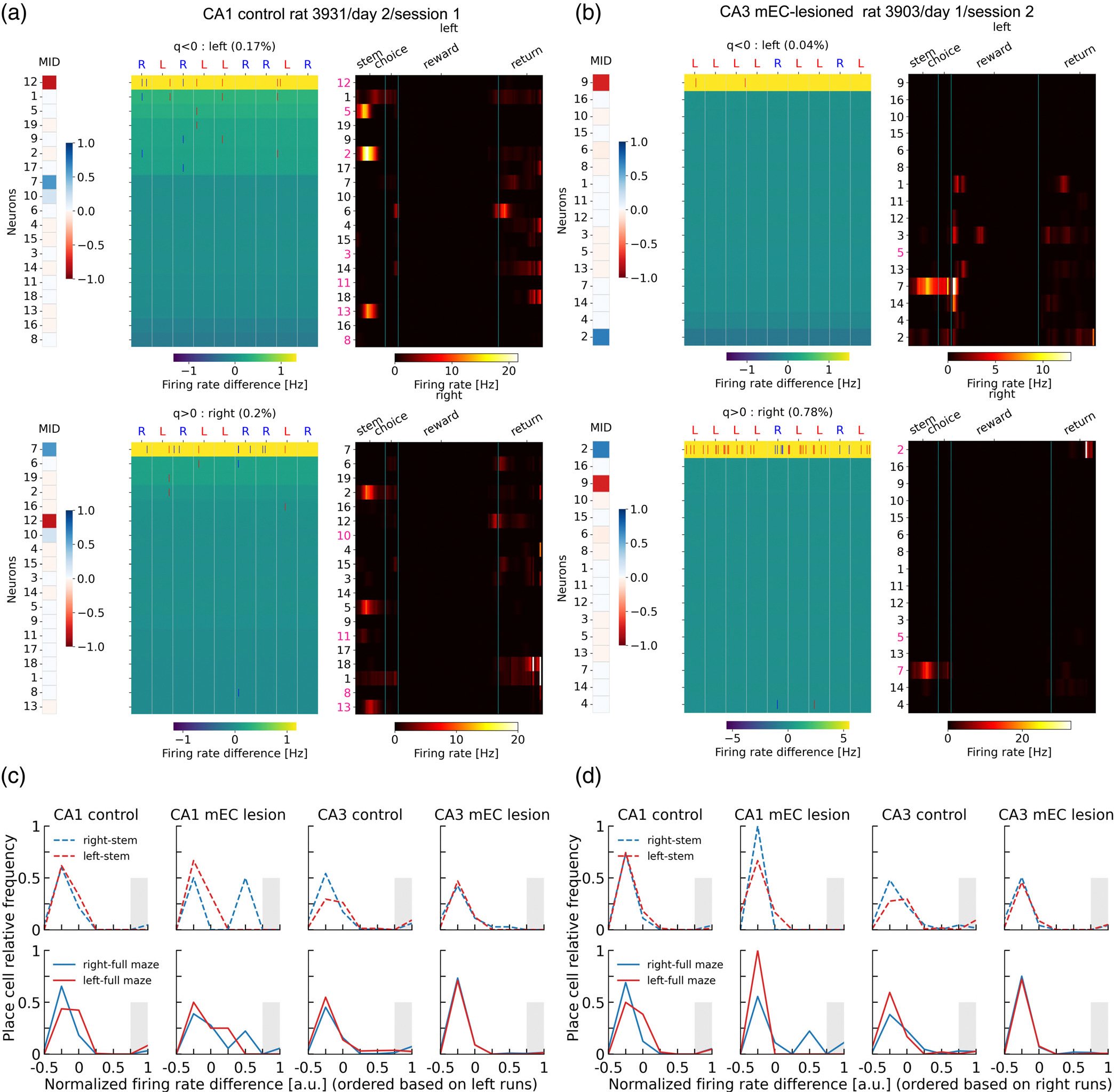
(a) For one example session, the MID (left) and raster plots (as in Figure 4c) are plotted with cells ordered according to the firing rate difference between relevant and non-relevant time bins (background colors). Spatial firing fields averaged over all trials are plotted on the right. Analysis is repeated separately for left (top) and right (bottom) runs. (b) Same as (a) for a second example session. (c,d) Place field propensity pooled over all sessions. (c) The fraction of cells with significant place fields on the stem (top) and on the whole maze (bottom) is plotted as a function of the normalized rate difference of a cell’s delay activity between relevant and non-relevant bins from leftward runs (color code in a and b top). (d) Same as (c) with rate difference determined from rightward runs (color code in a and b bottom). A cell was considered a significant place cell if the spatial information (Skaggs et al., 1993) exceeded the 95%ile of the null distribution obtained from shuffled spiking activity. Gray bars in (c), (d) indicate the interval 0.8–1.0. Two sided ranksum tests were performed on whether the difference between red and blue lines in the gray box was significantly different between left ordering (c) and right ordering (d). No test was significant with all *p*-values larger than 0.55.

Finally, we were asking if delay period activity during relevant patterns is connected to specific place field firing along the figure 8 maze. Specifically, it would be interesting whether place cells on the stem arm of the maze would show a correlation between activation in relevant time bins during the delay period and splitter cell activity (Wood et al., 2000). We therefore re-computed place fields of the rat data sets from (Sabariego et al., 2019) for left- and right-ward runs independently. Place cell activity was then visualized with the cell indices ordered according to the firing rate difference (color code) between relevant and non-relevant bins in the delay phase (Figure 6a,b). Visual inspection showed no relation between high (or low) firing in relevant bins and place field location. We therefore computed the fraction of places cells as a function of the normalized firing rate difference between relevant and non-relevant time bins for place fields on the stem (Figure 6c,d top) and all place fields (Figure 6c,d bottom). Most place cells exhibited a slightly reduced activity in the relevant bin (peaks are at slightly negative normalized rate differences). A small subset of place cells, however, is recruited from cells with maximum normalized rate differences, at least in the control populations. We thus asked, whether these cells have splitter characteristics and tested the difference of relative place cell frequencies in the interval 0.8–1.0 normalized firing rates (gray bars) between left run-ordered and right run-ordered rate differences. If the cells with high rate differences were splitter cells, one would expect that the difference between rightward and leftward fields would be smaller when ordered according to right-ward rate difference as compared the left-ward rate difference, since a delay activity upon a previous left/rightward run would be more predictive for a right/leftward field in the next run. Conversely, we would expect that the difference between rightward and leftward fields would be larger when ordered according to right-ward rate difference as compared the left-ward rate difference, if delay activity upon a previous left/rightward run would be reflecting a left/rightward field reverberation from the previous run. Ranksum tests between differences of relative place cell frequencies, however, were not significant in either direction and in either of the four animal cohorts, independently of whether we considered only place fields on the stem or place fields on the whole maze (for details on the test, see caption of Figure 6).

We therefore conclude that the informative activity in the delay pattern does not reflect spatial or task-related activity on the maze, but more likely reflects an internal network state that may or may not be transferred to downstream regions directly but could maintain short-term memory information in the local network as an after effect.

## 4 | DISCUSSION

We examined the short-term memory content of hippocampal CA1 and CA3 activity for rats during a delayed spatial alternation task and CA1 activity for Mongolian gerbils, after navigating virtual reality mazes, during reward consumption in inter-trial intervals. The recorded activity of past experience was decoded applying a linear neural network to population vectors from time bins of length of 100 ms. To directly identify the neuronal basis of the prediction accuracies we visualized the decision boundary using adversarial attacks, and subsequently identified most informative neuronal ensembles in terms of vector clusters orthogonal to the decision boundary. Applying these neuronal ensembles to the recorded activity we were able to extract the activity patters most related to previous behavior. Few neurons (about 20% of those that were active in the delay periods) and few time bins (2%) seem to be contributing to the classification task with relatively low firing rates (about 2.5 Hz) across all experimental conditions. We reasoned that this may indicate that the recent past is encoded with sparsely dispersed spikes. Since the amount of informative activity patterns was reduced at least in CA3 of lesioned rats, our results suggest that this low-rate activity correlate with working memory processes.

The mEC provides the hippocampus with spatial information during foraging and navigation tasks (Gil et al., 2018; Hafting et al., 2005). Previous analysis observed that mEC is necessary for control level working memory performance (Sabariego et al., 2019), despite only limited effects on hippocampal place fields (Hales et al., 2014; Schlesiger et al., 2015) and sequence replay (Chenani et al., 2019). Our results suggest an additional mEC-dependent mode of activity that appears to hold information related to previous experience, which entails a relatively low number of active cells and spikes. A further decline of predictivity was observed in gerbils navigating through virtual environments with conflicting sensory-motor context, suggesting that particularly sensory information via the mEC may be a main driver for the informative low rate activity patterns.

Because the observed brief periods of informative activity are sparse and random in time, potential mechanisms that may give rise to them are unlikely to consist of local persistent neural firing generated by positive self-feedback (Fransén et al., 2006). Synfire chains (Abeles, 1991) that propagate through multiple brain areas, however, cannot be excluded, but would require that similar temporally correlated activity signatures would be visible in other limbic brain areas. Particularly the medial prefrontal cortex with its direct hippocampal innervation, however, may lack such informative activity (Böhm & Lee, 2020). An alternative mechanism to store short-term memory is synaptic short-term dynamics (Leibold et al., 2008; Mongillo et al., 2008). The synapse-specific depression and facilitation states may maintain specific behavioral information for time scales up to few seconds, however, these states would need to be refreshed every few seconds to bridge intervals of several tens of seconds as in the currently investigated behavioral tasks. The low rate activity patterns described in this paper may implement such a refreshing mechanism.

Classifier-based decoding is a robust method to link specific features of neuronal activity to cognitive function and behavior. The existence of several well-tested classifier implementations that are straight-forward to analyze within the theoretical framework of hypothesis testing and cross validation is particularly convenient (Bishop, 2006) and makes them good candidates for decoding typically low signal to noise neuronal activity (Haynes & Rees, 2006; Lemm et al., 2011; Norman et al., 2006). The downside of classifier-based decoders is that they usually come as a black box, meaning that the neuronal activity features which are the most influential regarding a specific behavioral outcome are not readily observable. However, knowing these features, is pivotal for correlating neuronal ensembles to behavioral states. Here, we employ explainable artificial intelligence methods (Karimi et al., 2019), known as adversarial attacks, in order to sample the decision boundary of classifiers in an attempt to overcome their black-box nature (Doran et al., 2017). In doing so, we identify the most informative neuronal ensembles in terms of consistently appearing clusters of normal vectors relative to the decision boundary.

Classification of high-dimensional (multi-neuron) data with low signal to noise ratio and limited numbers of trials is usually best done with linear models, since more complex non-linear classifiers are prone to overfitting, exhibiting test performances that are drastically inferior to training performances (Bishop, 2006). Although linear classifiers may thus turn out superior in many of the real-world applications from an empirical risk minimization perspective, the true underlying generative processes may nevertheless be non-linear. Our attack-based approach provides a handle to uncover at least parts of the underlying non-linearities with a linear network, by multiple subsamplings for each of which we estimate the normal vector. Clustering of normal vectors from the many subsamplings and applying consistency measures allows to detect multiple considerably distinct clusters of normal vectors, and thus allows to effectively describe some of the non-linear structure in the data.

One of the limitations of a multivariate classifier approach is that the statistical power is limited by the number of trials or the length of a recording session. For example, a small significant rate difference, was found in CA1 and CA3 of control rats in the first 5 s of the delay period in Sabariego et al. (2019) using the grand average of all single units over all sessions. Our multivariate method did not detect such a small rate effect (Figure 5b), but rather is tuned towards finding information in the covariance structure of the activity.

How to maintain information over time intervals of tens of seconds to minutes, and how to achieve this at low energetic costs, are key open problems in understanding the cortical basis of working memory. Particularly the energy constraint will restrict the neuronal activity correlates to be sparse and low-rate, properties that make them hard to find. Further new analysis approaches will be needed to identify such neural signatures, particularly also in correlation with behavioral measures (Schneider et al., 2023).

## Supplementary Material

Supporting Information

## Figures and Tables

**TABLE 1:** Session statistics.

Condition	Min	Max	Mean

(i) % of inactive cells			
CA1 control	0.00	33.30	7.72
CA1 mEC lesion	0.00	33.30	5.56
CA3 control	0.00	75.00	16.25
CA3 mEC lesion	0.00	24.56	6.23
CA1 LR maze	0.00	0.00	0.00
CA1 L*R* maze	0.00	2.94	0.16
(ii) Trials count			
CA1 control	8.00	10.00	8.88
CA1 mEC lesion	8.00	10.00	8.63
CA3 control	8.00	10.00	8.93
CA3 mEC lesion	8.00	10.00	8.81
CA1 LR maze	20.00	20.00	20.00
CA1 L*R* maze	20.00	20.00	20.00
(iii) Test patterns count			
CA1 control	2400	3000	2667
CA1 mEC lesion	2400	3000	2588
CA3 control	2400	3000	2678
CA3 mEC lesion	2400	3000	2642
CA1 LR maze	2000	2000	2000
CA1 L*R* maze	2000	2000	2000

**TABLE 2:** Test statistics, *p* values, degrees of freedom (*df*) for Figure 2a.

(i) CA1				

Rat/day/sess.	3906/1/1	3906/1/2	3931/2/1	3931/3/2

ANOVA (*F*)	1.08, 0.38	1.75, 0.093	1.93,0.061	7.16, 1.6e–8
*T*	0.48, 0.64	2.05, 0.041	2.1, 0.032	1.50, 0.13
*S*	−1.11, 0.27	−1.38, 0.17	1.35, 0.18	−0.50, 0.62
*M*	−0.15, 0.89	0.34, 0.74	0.68, 0.50	−0.90, 0.37
*F*	−1.13, 0.26	−1.42, 0.16	0.44, 0.66	−1.78, 0.075
*R*	1.91, 0.056	1.18, 0.24	−1.36, 0.17	6.53,7.7e–11
*v*	0.98, 0.33	0.88, 0.39	1.68, 0.093	−1.61, 0.11
*R/L*	−0.10, 0.92	0.19, 0.86	−0.08, 0.94	0.24, 0.82
*df*	4774	5381	4779	3584

(ii) CA3					

Rat/day/sess.	3839/3/2	3931/2/2	3931/3/2	3958/1/2	3958/3/1

ANOVA (*F*)	0.51, 0.83	1.40, 0.21	1.50, 0.17	4.88, 1.7e-5	1.57, 0.14
*T*	−0.66, 0.52	1.27, 0.21	−2.44, 0.015	−1.90, 0.059	0.39, 0.70
*S*	−0.64, 0.53	−0.62, 0.54	−1.31, 0.20	0.00, 1.00	−1.97, 0.050
*M*	0.59, 0.56	2.33, 0.020	−1.00, 0.32	−1.45, 0.15	−0.07, 0.95
*F*	1.03, 0.31	0.25, 0.81	1.37, 0.18	0.58, 0.57	−0.88, 0.39
*R*	−0.04, 0.97	−1.25, 0.22	0.65, 0.52	−3.92, 9.0e-5	−1.70, 0.089
*v*	0.88, 0.39	1.19, 0.24	−0.98, 0.33	1.90, 0.058	−0.58, 0.57
*R/L*	−0.16, 0.88	0.11, 0.92	−0.02, 0.99	0.06, 0.96	−0.22, 0.83
*df*	4784	5383	3584	4782	3572

**TABLE 3:** Test statistics (*N, df*) for Figure 2b.

Regressor	1	*T*	*S*	*M*	*F*	*R*	*v*	*R/L*	

CA1 (black), rank	1	10	4	5	1	8	6	7	
CA1 (black), *N*									4
CA1 (red), *T*	−2.06	3.59	−0.76	0.27	−2.09	3.37	1.46	0.01	
CA1 (red), *df*									18,542
CA3 (black), rank	12	4	1	7	12	2	10	5	
CA3 (black), *N*									5
CA3 (red), *T*	1.96	−1.50	−1.64	0.22	1.44	−1.76	1.00	−0.03	
CA3 (red), *df*									22,137

**TABLE 4:** Test statistics, *p* values, degrees of freedom (*df*) for Figure 5c–h.

Condition	Test statistic	*p* values	Degrees of freedom (*df*)

(i) Figure 5c, Wilcoxon test, median >0.5			
CA1 control	32.394	3.28e–230	*n* = 2127
CA1 mEC lesion	−1.697	.090	*n* = 25
CA3 control	24.968	1.37e–137	*n* = 3215
CA3 mEC lesion	9.7334	2.164e–22	*n* = 161
CA1 LR maze	23.615	2.714e–123	*n* = 939
CA1 L*R* maze	8.033	9.50e–16	*n* = 120
(ii) Figure 5c, Mann-Whitney *U* test			
CA1 control	11.276e7	1.316e–84	*n*_1_ = 2127, *n*_2_ = 86685
CA1 mEC lesion	10.304e4	.2	*n*_1_ = 25, *n*_2_ = 9575
CA3 control	26.615e7	1.705e–16	*n*_1_ = 3215, *n*_2_ = 153987
CA3 mEC lesion	72e5	5.762e–05	*n*_1_ = 161, *n*_2_ = 103639
CA1 LR maze	46.367e6	2.340e–67	*n*_1_ = 939, *n*_2_ = 74943
CA1 L*R* maze	62.289e4	4.24e–10	*n*_1_ = 120, *n*_2_ = 7837
(iii) Figure 5d, Mann-Whitney *U* test			
CA1 control	290.0	5.473e–05	*n*_1_ = 18, *n*_2_ = 18
CA1 mEC lesion	4.0	.333	*n*_1_ = 2, *n*_2_ = 2
CA3 control	766.0	3.092e–06	*n*_1_ = 30, *n*_2_ = 30
CA3 mEC lesion	356.0	3.213e–07	*n*_1_ = 19, *n*_2_ = 19
CA1 LR maze	272.0	.008	*n*_1_ = 19, *n*_2_ = 19
CA1 L*R* maze	3.0	.667	*n*_1_ = 2, *n*_2_ = 2
(iv) Figure 5e, Mann-Whitney *U* test			
CA1 control	294.0	3.168e–05	*n*_1_ = 18, *n*_2_ = 18
CA1 mEC lesion	4.0	.333	*n*_1_ = 2, *n*_2_ = 2
CA3 control	833.0	1.554e–08	*n*_1_ = 30, *n*_2_ = 30
CA3 mEC lesion	361.0	1.443e–07	*n*_1_ = 19, *n*_2_ = 19
CA1 LR maze	291.0	.001	*n*_1_ = 19, *n*_2_ = 19
CA1 L*R* maze	3.0	.667	*n*_1_ = 2, *n*_2_ = 2
(v) Figure 5f, Wilcoxon test, median > 0.5			
CA1 control	4.541	5.608e–06	*n* = 74
CA3 control	12.478	9.824e–36	*n* = 527
CA3 mEC lesion	1.842	.065	*n* = 53
CA1 LR maze	14.784	1.860e–49	*n* = 173
(vi) Figure 5f, Mann-Whitney *U* test			
CA1 control	97729.5	.030	*n*_1_ = 74, *n*_2_ = 2326
CA3 control	5668054.0	2.423e–4	*n*_1_ = 527, *n*_2_ = 19753
CA3 mEC lesion	121469.0	.006	*n*_1_ = 53, *n*_2_ = 5827
CA1 LR maze	358196.0	4.572e–39	*n*_1_ = 173, *n*_2_ = 2627
(vii) Figure 5g, Mann-Whitney *U* test			
CA1 control	25	.08	*n*_1_ = 5, *n*_2_ = 5
CA3 control	830.0	.117	*n*_1_ = 37, *n*_2_ = 37
CA3 mEC lesion	86.0	.101	*n*_1_ = 11, *n*_2_ = 11
CA1 LR maze	38.0	.097	*n*_1_ = 7, *n*_2_ = 7
(viii) Figure 5h, Mann-Whitney *U* test			
CA1 control	25	.08	*n*_1_ = 5, *n*_2_ = 5
CA3 control	1248.0	1.153e–9	*n*_1_ = 37, *n*_2_ = 37
CA3 mEC lesion	121.0	8.152e–5	*n*_1_ = 11, *n*_2_ = 11
CA1 LR maze	27.0	.798	*n*_1_ = 7, *n*_2_ = 7

## Data Availability

All behavioral and electrophysiological data is available from the lead contact upon request. Additionally, all original code (usable in Python 3) is available at https://github.com/MarkosAthanasiadis/patternsfromattacks. Any additional information required to reanalyze the data reported in this work paper is available from the lead contact upon request.
