# Supplementary material for: Low rate hippocampal delay period activity encodes behavioral experience
Source: Hippocampus. Author manuscript; Available in PMC 2025 Apr 8. (PMC11978360; doi:10.1002/hipo.23619)
Supplement: Supporting Information [file NIHMS2065343-supplement-Supporting_Information.pdf]

Low Rate Hippocampal Delay Period Activity Encodes Behavioral Experience

Athanasiadis et al.

April 8, 2024

Supporting information

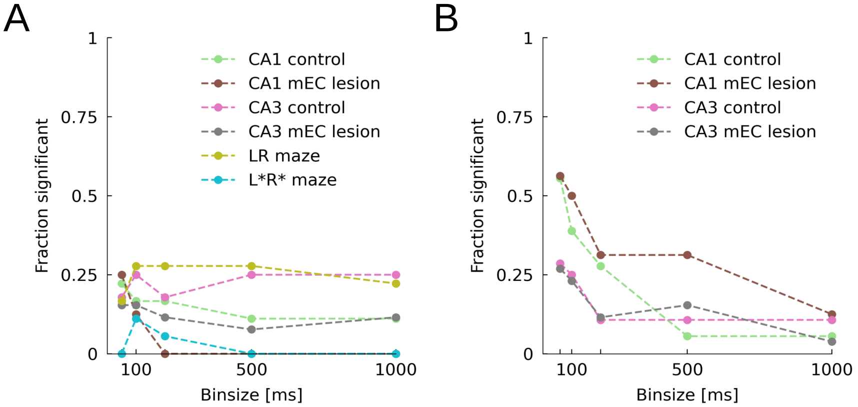

Figure S1: Robustness of decoding. (A) The fraction of significantly decodable sessions is shown as a function of the size of the time bins used to construct population vectors (same as Figure 1E of the main paper) obtained with a linear support vector classifier instead of a neural network. The outcomes are similar to Figure 1E. (B) Same as A using a trial-based rate normalization (as used in Sabariego et al., 2019) shows a reduction of decoding performance for long time bins, indicating that, on long time scales sizes, information is encoded in firing rate rather than covariance structure.
